# Supplementary material for: Are carbon emissions trading and green financial instruments synergistic? -Comprehensive quantitative research based on content analysis
Source: PLoS One. 2024 Mar 7;19(3):e0298601. doi: 10.1371/journal.pone.0298601 (PMC10919601; doi:10.1371/journal.pone.0298601)
Supplement: S1 Data — (ZIP) [file pone.0298601.s001.zip › Data/地级市Z_F工作报告.zip!/╜¡╬≈/╔╧╚─╩╨/└┤╫╘╣1⁄2╓┌║┼í╛┬φ┐╦╩2╛▌═°í┐_2008.docx]

各位代表：

现在，我代表市人民政府向大会作工作报告，请予审议，并请市政协各位委员和列席会议的同志提出意见。

一、过去一年的工作回顾

2007年是上饶推进科学发展、和谐发展的重要一年。在省委、省政府和市委的正确领导下，全市上下坚持以邓小平理论为指导，努力践行"三个代表"重要思想，全面落实科学发展观，抢抓机遇，扩大开放，锐意进取，奋力拼搏，全市经济社会呈现又好又快的发展势头，实现了"八个突破"：GDP总量突破500亿元，达到528亿元；人均GDP突破1000美元，达到1104.5美元；财政总收入突破50亿元，达到55.4亿元；财政占GDP的比重突破10%，达到10.5%；规模以上工业增加值突破100亿元，达到120亿元；城区居民人均可支配收入突破10000元，达到11643元；高速公路总里程居全省第一，实现县县通高速公路；实现县县财政收入超2亿元。

（一）坚持跨越发展，经济实力迅速增强

经济总量不断提高。预计全年地区生产总值增长17%（现价），财政总收入增长34.9%，其中地方财政收入32.1亿元，增长26.2%；完成全部工业增加值194.5亿元，增长29.6％；完成全社会固定资产投资405亿元，增长27.2％，其中城镇固定资产投资310亿元，增长25%；实现社会消费品零售总额186.4亿元，增长18％。

发展质量明显优化。财政总收入占GDP比重上升1.4个百分点，达到10.5%；税收占财政总收入比重提高4.2个百分点，达到84.4%；全部工业增加值占GDP比重上升3.6个百分点，达到36.8％；规模以上工业增加值占全部工业增加值的比重上升4.9个百分点，达到61.7％；外贸进出口总额占GDP 的比重上升1个百分点，达到4％。

县域经济整体推进。各县（市、区）的财政收入继2005年县县过亿元后，2007年又实现县县超2亿元。其中，广丰县达到10.3亿元，德兴市达到9亿元，横峰县财政收入实现两年翻一番，达到2.1亿元。乡镇财政实力显著增强，全市财政收入超1000万元的乡镇达到61个，占乡镇总量的三分之一。其中，德兴泗洲镇、广丰永丰街道办、余干黄金埠镇的财政收入均超亿元。

市本级财力快速壮大。2001年至2006年财政收入年均递增26.2%，2007年加快提速，同比增长49%，由2001年的1.3亿元增加到2007年的6.2亿元。其中，上饶经济开发区财政总收入达到7531万元，三管委财政总收入达到2090万元。

（二）坚持协调发展，三次产业相互促进

工业主导地位更加凸现。预计规模以上工业增加值增长40.8％，在全省排位由2006年的第8位上升到第7位，占全省的比重由2006年的6.4%上升为7.0%。全市园区（开发区）实际开发面积达到50.6平方公里，实现主营业务收入408亿元，增长66.6%，上交税金16.6亿元，增长67.7%。支柱产业作用明显加强，有色金属、机械、建材三大产业完成规模以上工业增加值72亿元，占规模以上工业总量的60%。实施"十百千亿工程"取得成效，新增规模以上工业企业87家，总数达到581家，新增年销售收入过亿元工业企业29家，累计达81家。

农业经济更具活力。农业经济总量稳步提升，预计全年完成农业增加值100亿元，增长5.7%。粮食生产再创新高，达到54.1亿斤，增长1.6%。农业产业化进程加快，新增农业企业179家，总数达2395家，新增规模以上农业企业70家，总数达324家；新增销售收入过亿元企业10家，总数达到16家，万年贡米集团年销售收入超过10亿元。农业标准化生产扎实推进，获江西省名牌农产品35个，占全省的33%；建立绿色食品原料标准化生产基地7个、无公害农产品基地104个，继续保持全省领先。成功举办首届农业产业招商暨农产品展销会，与上海浦东新区农业战略合作全面开展，农业经济外向度进一步提高。

第三产业更加繁荣。预计三产完成增加值195.5亿元。旅游产业不断壮大，全年接待国内旅客890万人次，增长17.1％，国内旅游收入50.5亿元，增长20.5％；入境旅游3.8万人次，增长26.3％。重点景区建设步伐加快，三清山风景名胜区申报世界自然遗产，已通过世界自然保护联盟专家实地考察评估，龟峰和婺源县分别被授予"世界地质公园"和"中国旅游强县"称号。商贸流通业迅速发展，新增规模以上市场（商场）37个，累计达到132个，新增营业面积94. 7 万平方米 ，增长67％，新增年销售收入过亿元的大型市场（商场）8家，累计达到33家。"万村千乡市场工程"扎实推进，新建农家店885家，总数达1772家，上规模的配送中心累计达9家。金融保险、邮电通讯、交通运输、烟草、石油、房地产等产业健康发展。

（三）坚持统筹发展，城乡面貌出现可喜变化

城市建设步伐加快。中心城市"一城三区"格局初步形成，规划控制面积扩大到160平方公里，建成区面积达到38.5平方公里，比2002年扩大了18.5平方公里。城市功能日益完善，市本级完成城市建设投资18.6亿元，市政基础设施投资5.8亿元。一批重点工程加快推进，中心区中央公园绿化工程全面启动，货场路、广信大道中段建成通车，凤凰大道西段路桥工程全部完成。市污水处理厂二期主体工程竣工并试运行，生活垃圾处理厂一期工程建成将投入使用，三江污水管网工程进展顺利。第一期148个公交候车亭和站牌建设全部完成。市图书馆、科技馆主体工程竣工，信江书院修缮工程顺利推进，奥林匹克中心完成了选址、立项和设计招标工作。全市城市绿化覆盖率达到38.3％，其中中心城区建成区绿地率为38.8%。全市城镇化率较去年提高2个百分点，达到31.5%。

新农村建设扎实推进。全年共投入新农村试点建设资金6.8亿元，1599个试点村建设全面完成。以中心城镇为重点的小城镇建设步伐加快。全年完成村镇建设投资26.5亿元，完成乡镇总体规划160个，开展村庄整治试点1520个。

交通网络逐步完善。预计全市完成交通基本建设投资30.8亿元。景鹰高速公路建成通车，上武高速公路的可行性研究报告通过交通部和国家发改委评审；昌德高速公路启动前期工作。全市高速公路总里程达到 381公里 。三清山（赣东）机场前期工作加快进行。滨湖地区渡改桥工程扎实推进，鄱阳古县渡大桥、莲湖大桥建成通车。完成农村公路硬化 1800公里 ，行政村路面硬化率达到63%。

农田水利设施不断夯实。病险水库除险加固累计开工747座，完工649座，验收通过491座；余干枫富联圩、鄱阳沿河圩除险加固工程进展顺利；11.9万人的饮水困难问题得到解决。实施造地增粮富民工程，造地4.8万亩。农业综合开发改造中低产田12万亩。

能源保障显著加强。完成"十一五"电力主网、城网、农网规划修编，黄金埠电厂一期2×65万千瓦机组并网发电，上饶第三回220千伏输变电工程建成投入运行，鄱阳220千伏输变电工程即将投入运行，上饶500千伏输变电工程完成50％工程量，今年6月份可投入运行。上饶经济开发区率先在全省完成工业园区"同网同价"试点工作，有效降低了企业投资成本。

信息化工作进展迅速。网上行政办公系统正式投入使用，全市政务信息网络基本建成，走在全省前列。农村信息化建设成效显著，被批准列入全国首批26个农村信息化综合信息服务试点市，率先在中西部地区实现100%自然村通电话、93%行政村通宽带，移动通讯信号行政村覆盖率达到100％，自然村覆盖率达到88％，基本实现50户以上自然村通移动电话目标。

（四）坚持创新发展，经济活力进一步增强

机制体制改革深入推进。全面完成林权制度主体改革，水管体制、畜牧兽医管理体制、种子管理体制改革顺利推进。朝阳磷矿争取政策性破产获得批准，医药商业企业划转地方政府管理全部到位，上饶肉联厂、上饶振大陶瓷公司进入依法破产程序，国有流通企业改制基本完成。成功组建上饶市商业银行。市直行政事业单位资产规范管理工作开始启动，全市机关事业单位工资制度改革套改审批工作基本完成，公务员规范津贴补贴工作平稳推进。

开放型经济水平明显提升。全年实际利用外资2.5亿美元，同比增长34.4％，引进内资374.9亿元，增长60.9%；预计实现外贸出口2.6亿美元，同比增长58％。上饶被国务院批准为开放城市；南昌海关上饶办事处正式组建；上饶"无水港"项目正式启动；网上招商平台"海峡西岸网洽会·上饶馆"正式开通。招商方式逐步实现由"招商引资"向"招商选资"转变，海螺水泥、晶科能源、梦娜袜业、金山矿业、金德铅业、婺女村旅游休闲度假区等一批投资10亿元以上的大项目落户上饶。

非公有制经济不断壮大。净增非公有制企业和个体工商户5708个，达到12.3万个，上缴税金24.4亿元，增长47.7％。其中非公有制工业企业完成增加值165.1亿元，增长38.6%，占全部工业增加值的84.9%，比上年提高11.6个百分点。

（五）坚持可持续发展，环境质量进一步提升

环境保护得到加强。环评力度进一步加大，对422个项目进行了环评审批，对226个违规投产建设项目下达了限期整改通知书，否决了77个投资规模小、工艺落后、设备陈旧、选址不合理的项目，关停取缔违法企业126家，限期治理98家。节能减排工作取得明显成效，淘汰了20万吨落后炼钢产能和59万吨落后水泥产能，整顿关闭了44对煤矿矿井，对上饶永利资源综合利用热电有限公司、江冶铅炼分厂实行了关停，全年减排化学需氧量1955吨，减排二氧化硫3112吨。完成水土流失综合治理253平方公里。

生态建设取得实效。绿色创建工作扎实推进，全市完成造林54.9万亩。饮用水源保护进一步加强，12个县（市、区）全部完成饮用水源一、二级保护区划定，并取缔中心城区饮用水源一级保护区内排污口3个，二级保护区内排污口6个。全市地表水水质断面基本达到国家Ⅱ类标准，水质达标率为100％。市中心城区二氧化硫、可吸入颗粒物均达到国家二级标准，二氧化氮达国家一级标准，城区环境空气质量优良率达100%。"五城同创"扎实推进，市本级和广丰、玉山、万年、德兴、婺源、上饶县等6个县（市）的县城获得了"省园林城市"称号。婺源江湾镇被命名为"全国环境优美镇"，实现了我省"环境优美乡镇"零的突破。

（六）坚持全面发展，社会各项事业扎实推进

科技事业取得新成绩。全年上报申请专利182件，有3项科技成果达到国际先进水平。新增江西名牌产品5个，累计达到27个。有2个项目列入国家发改委高技术产业化扶持项目计划，获专项扶持资金1250万元。有4个项目列入全省高新产业重大项目计划，获专项扶持资金1850万元。成功举办有色金属精深加工同舟论坛。

教育事业呈现新气象。全年县以上财政投入近3亿元用于义务教育阶段免学杂费、保障公用经费、补助寄宿生生活费及免费提供教科书；投入1.5亿元，新建改造校舍近20万平方米，购置课桌椅、讲台55.7万套；投入6800多万元，完成了全市2680所农村中小学远程教育工程。"两基"成功通过国家督导检查。高等教育质量进一步提高，职业教育发展加快，职校在校学生近5万人，增长13.6%。

文化体育广播事业实现新突破。荣获全国"节庆中华"示范城市称号，广场文化、社区文化、企业文化、乡村文化活动蓬勃开展，成功拍摄励志故事片《网络妈妈》，成功举办了上饶市首届农民艺术节。婺源县理坑村、汪口村被国家文物局列入"世界文化遗产"预备名单。成功举办环沪港国际自行车大赛上饶环城赛、省第三届全民健身运动会门球赛等10多项大型群众体育赛事，扎实推进了全民健身运动。电视转星工作全面完成。

卫生事业有了新进步。全市12个疾控国债项目建设和农村卫生院危房改造项目全部完成，公共卫生信息网络和食品药品安全信息网全面开通，市麻风病康复中心立项建设，重点传染病防治工作有效开展。血防工作扎实推进，共查血防病人26.9万人次，治疗病人14万人次。

人口和计划生育工作开创新局面。全市人口出生率为13.71‰，同比下降0.01个千分点，人口自然增长率为8.02‰，计划生育率同比上升8.5个百分点，实现了人口控制目标。

平安上饶建设迈上新台阶。加强信访工作，扎实开展矛盾纠纷排查和调处，全年未发生一起因矛盾纠纷激化而引起的重大群体性事件和群众性纠纷械斗。不断加大公共安全投入，严厉打击各类犯罪活动，社会治安明显好转，人民群众的安全感进一步增强。深入推进安全生产专项整治活动，交通、消防、煤矿、非煤矿山等重点领域安全生产形势平稳，全市未发生特大安全事故。食品药品安全形势持续好转，农村药品供应网络和监督网络基本建立，全年没有发生大的食品药品安全事故，社会保持和谐稳定。围绕经济建设中心，新闻出版、地方志、档案、保密、民族、宗教、外事侨务、涉台事务、气象服务、防震减灾、国防教育、人民防空、民兵预备役和"双拥"工作，以及社会福利、妇女儿童、老龄、残疾人、慈善等事业取得了新成绩。

（七）坚持和谐发展，改善民生取得突破

按照省政府的要求和部署，我市大力推进民生工程。年初确定的就业和再就业、社会保障、社会救助、医疗保障、济困助学、住房、扶贫开发、改善群众生产生活条件等关系民生的八个方面40件实事全部得到落实。全市城镇新增就业人数6.7万人，新增转移农村劳动力11.2万人；43.4万城镇职工参加基本养老保险，3万农民工参加养老保险。17.9万农村困难群众享受农村低保，11.8万城镇困难群众享受城市低保。41.5万城镇职工参加基本医疗保险，436万农民参加新型农村合作医疗。有22.2万农村困难群众、12.9万城镇困难群众得到大病医疗救助，发放救助资金5393万元。93万义务教育阶段的学生被免除学杂费，24万贫困家庭学生得到免费提供的教科书，8600多名普通高中学生、6100多名中等职业教育学生、1800多名考入大学的贫困家庭学生得到资助。城镇居民基本医疗保险参保人数达到34万人，我市被列为全国79个城镇居民基本医疗保险试点城市之一。移民扶贫搬迁进展顺利，6500名山区、湖区、库区贫困群众春节前将全部搬进新居。新增农村沼气2.5万户，保有量达到11.3万户。全市经济适用房开工建设46. 6 万平方米 ，总投资3.3亿元，已有1599户获得购买资格；廉租房受益总户数2034户。部分群众就业难、读书难、看病难、住房难等问题得到有效解决。

经济增长和民生工程投入的增加，让广大人民群众得到了更多的实惠。城区居民人均可支配收入增长18.4%；农民人均纯收入达到3900元，增长10.7%。

（八）坚持依法行政，政府自身建设进一步加强

加强民主法制建设，自觉接受人大及其常委会法律监督、工作监督和政协民主监督，重视民主党派和社会各界的参政议政作用，认真办理人大代表建议和政协提案，办结率达到100%，满意率达到94.5%。扎实开展普法教育，认真实施《公务员法》，加强规范性文件管理，对2000年以来市政府及市政府办公室下发的所有规范性文件进行了审核、清理，修改和停止执行了部分文件。切实落实决策听证、重大决策专家咨询、公众建议征集等制度，进一步加强市长热线工作，民情通道更加畅通，依法决策、科学决策、民主决策水平进一步提高。开展了应急演练，应急处理能力进一步增强。扎实开展"加强作风建设,实现新的跨越"集中教育活动和政风行风评议活动，荣获全省政风行风评议综合排位第一名。更加重视廉政建设，强化行政监察、审计和经济监督，政务环境进一步改善，投资环境进一步优化，荣获2007"世界投资中国-中小城市魅力奖"，再次被评为"浙商（省外）最佳投资城市"。

一年来的成就，是省委、省政府和市委正确领导的结果，是全市广大干部群众辛勤劳动、共同奋斗的结果。在此，我谨代表市人民政府，向全市人民和驻饶人民解放军、武警官兵、公安干警和中央、省驻饶企业，向给予政府工作支持和监督的人大代表、政协委员、各民主党派、工商联、无党派人士和各人民团体，向关心和支持上饶建设的海内外朋友，向广大老干部、老同志和关心家乡发展的在外乡友，向在上饶创业的境内外投资者、合作者，表示衷心的感谢！

总结一年来的工作，我们也清醒地认识到，上饶经济社会发展中还面临许多亟待解决的困难和问题。主要是：经济总量仍然较小，质量还有待提高，结构还需进一步优化，转变经济发展方式的任务还十分艰巨；人口、资源、能源的压力较大，节能减排形势比较严峻；农业产业化水平还不高，农业基础地位还有待巩固；受土地报批、征地拆迁、资金调度及规划调整等因素制约，年初确定的部分城建重点工程进展不够理想；涉及人民群众切身利益的问题还比较突出，改善民生工作任务相当繁重；社会管理和公共服务领域还存在不少问题，政府及其部门的服务水平还有待进一步提高。对这些问题，我们将予以高度重视，不断在发展中切实加以解决。

二、2008年的工作思路

今年是全面贯彻落实党的十七大精神的第一年，也是我市全面建设小康社会极其重要的一年。政府工作的指导思想是：以党的十七大精神为指导，深入贯彻科学发展观，坚持好字优先，着力转变经济发展方式，优化经济结构和提高经济增长质量，深入推进改革开放和自主创新，切实加强节能减排和生态文明建设，更加重视改善民生和促进社会和谐，努力推动经济社会又好又快发展。

综合考虑各种因素，2008年全市经济社会发展主要预期目标是：生产总值增长15%，达到608亿元；财政总收入增长21%，达到67亿元，力争增长26.4%，达到70亿元；全社会固定资产投资增长20%，达到486亿元；社会消费品零售总额增长18%，达到220亿元；城镇登记失业率控制在3.48%以内；城区居民人均可支配收入和农村居民人均纯收入分别增长9%和7.7％;人口自然增长率力争控制在8‰以内；城镇化率提高2个百分点；每万元生产总值能耗、电耗、化学需氧量、二氧化硫排放量严格控制在省政府下达的指标之内。

按照上述指导思想和主要目标，新的一年，我们将重点抓好以下工作：

（一）不断壮大综合经济实力

加快发展，不断壮大经济实力，始终是上饶当前乃至今后相当长一段时期政府工作的首要任务。要进一步增强发展意识，把握发展规律，创新发展理念，转变发展方式，破解发展难题，提高发展质量，实现又好又快发展。

1、以新农村建设为重点，促进农村经济全面发展

做大农业经济总量。进一步增强做好"三农"工作的紧迫感和责任感。全面落实中央有关支农惠农政策，充分发挥广大农民种粮的积极性，不断增加农业投入，切实加强农业基础建设，提高农业综合生产能力，促进粮食稳定增产，确保粮食总产量达到52亿斤，力争达到54亿斤，全市农业增加值增长6%，达到106亿元。

大力发展现代农业。创新"三农"工作思路，转变农业发展方式，围绕大米、水产、生猪、茶叶、油茶、杨树、毛竹等特色优势产业，加快推进农业产业化，着力抓好农产品加工转化，培植一批主营业务收入超亿元的农业产业化龙头企业，力争新增规模以上农业企业50家。加快农业标准化进程，建设生猪、茶叶、红芽芋、特种水产、食用菌等大型优质农产品生产基地。规范建设一批"一村一品"示范村和农民专业合作社，引导各类市>主体参与农业产业化经营。

抓好新农村试点村建设。加紧做好乡村区域布局规划、新农村建设总体规划和村庄整治建设规划。以中心城区、城镇、中心集镇周边的村庄为重点，整合各类资金，改善农村基础设施建设，扎实推进1300个新农村试点村建设。

2、以大项目建设为重点，提高工业经济发展水平

坚定不移地实施主攻工业战略，大力推进新型工业化进程，力争规模以上工业增加值增长25％以上，总量突破160亿元；主营业务收入增长30％以上，总量突破500亿元；利税总额增长30％以上，总量突破35亿元。

做特做优工业园区。不断创新工业园区建设与管理模式，进一步加快园区基础设施建设。建立健全项目转移利益分享机制，提高园区的产业集群水平。推进节约用地、集约用地，提高单位土地投资强度和产出效益，提升园区整体档次，实现园区"二次创业"。力争主营业务收入超150亿元的一类工业园区达到2个，超40亿元的二类工业园区达到6个。

做大做优支柱产业。突出抓好有色金属、光学电子元件、客车机械制造、轴承、新型干法水泥、新型墙材、大米加工、食品添加剂等特色产业基地建设。重点推进以金德铅业、金山矿业、华丰铜业为龙头的有色金属产业，以凤凰光学、鑫新股份和富达铜材为龙头的机械产业，以万年青水泥、海螺水泥为龙头的新型建材产业，以翁福化工为龙头的硫化工产业，力争主营业务收入分别突破200亿元、60亿元、50亿元和10亿元。

做大做强重点企业。通过技术创新、产业扩张、资产重组、企业上市等多种途径，着力培育壮大一批强而大的优势企业。加快中金吉斯曼金山矿业有限公司日处理5000吨矿石项目、金德铅业20万吨铅冶炼技改项目、香港世华3万台汽车底盘生产线、新加坡三达膜集团碳素项目、梦娜袜业、欧美意鞋业等项目建设，确保年内竣工投产。支持上饶博能客车厂引进国际战略合作者，做优上饶客车产业。加快建设以江西月兔硅实业有限公司为龙头的高纯度石英粉基地，形成1.2万吨产能，并通过上下游配套，发展光伏产业。做大做强汉玉王陶瓷、德兴百勤集团的异VC纳和维生素B项目。切实抓好鄱阳格林艾文风电设备产业和列入国家"863"计划的婺源新型磁性材料项目建设。力争新增规模以上企业100家，年销售收入过亿元企业突破100家。

3、以扩大消费为重点，加快发展服务业

全面提高旅游业竞争力。充分发挥我市旅游资源优势，统筹整合自然、历史、人文等旅游资源，大力发展生态旅游、文化旅游、红色旅游，积极发展"农家乐"、"渔家乐"等休闲度假旅游和乡村旅游，加快发展入境游。不断提升精品旅游区管理水平，加快新旅游区建设步伐。确保三清山申报世界自然遗产圆满成功，扎实做好龟峰丹霞地貌、婺源理坑、汪口徽派建筑申遗的准备工作。力争全年实现旅游总收入60亿元。

加快发展现代服务业。启动中心城区粮油市场建设，开工建设赣东北万福农产品物流配送中心，完成花卉苗木交易市场建设。积极发展商贸、餐饮、社区服务业。抓好中心城区亿升广场等重点商业区项目建设。不断完善城乡市场体系。深入推进"万村千乡市场工程"，新建农家店900个、上规模的配送中心2家。大力发展金融、保险、信息业。加快发展证券、咨询、中介等新兴服务业。抓好以招商引资、项目推介、商品展销为主题的节庆和会展经济。推进房地产业健康发展。

强化城乡市场监管。继续巩固产品质量和食品安全专项整治成果，不断完善全市产品质量和食品安全长效机制。围绕食品、药品、农资、建材等与人民群众生产生活密切相关的重要商品，进一步加大打假执法力度，有效净化市场环境。进一步加大各类专项整治力度，有效规范市场经济秩序。进一步健全应急机制，提高市场监管应急处置能力，及时消除影响，保障供给，维护市场稳定，营造和谐的消费氛围。

（二）扎实推进民生事业

民生是经济发展的出发点和落脚点。今年，我们将更加注重保障和改善民生，加快实现城乡困难群众最低生活保障全覆盖，城乡困难群众大病医疗救助全覆盖，城乡义务教育免学杂费和贫困生助学全覆盖，农民和缺乏基本医疗保障的城镇居民合作医疗全覆盖，城市特困群众廉租住房应保户全覆盖等"五个全覆盖"，努力使全市人民学有所教、劳有所得、病有所医、老有所养、住有所居。重点抓好8个方面46件实事。

1、继续做好就业和再就业工作。着力办好6件实事：一是抓好1.55亿元小额担保贷款发放。二是做好下岗失业人员、城镇登记失业人员、未就业高校毕业生、城镇复员退伍军人的免费技能培训和创业指导工作。三是抓好城镇零就业家庭就业工作，确保有能力和有就业愿望的零就业家庭至少有一人就业。四是对1000名城乡残疾人进行职业培训，并为残疾人购买公益性岗位。五是对安置残疾人就业的福利企业，按照标准予以补助。六是推进农村富余劳动力转移就业，完成转移培训10.7万人,新增农村劳动力转移7.9万人。力争完成城镇新增就业人员4.7万人。同时，认真抓好《劳动合同法》、《就业促进法》贯彻落实。

2、切实强化社会保障。着力办好3件实事：一是扩大城镇职工养老保险覆盖面，争取参保人数达到48.9万人。二是提高城镇企业退休人员基本养老金水平，月人均增加100元。三是资助农垦企业务农职工参加基本养老保险。四是扩大工伤保险覆盖范围，力争职工参保23万人、农民工参保3.5万人。

3、进一步提高社会救助水平。着力办好8件实事：一是扩大农村低保覆盖面，适当提高低保发放水平，农村低保人数由17.4万人扩大到26万人，月人均补差提高到35元。二是城市低保补差由每人每月85元提高到95元。三是提高农村五保对象集中供养率。四是完善城乡临时救助制度，提高对因临时性、突发性事件造成生活困难群众的救助能力。五是提高国有"农林水"困难企事业单位中未参加养老保险、且基本生活保障水平低的退休职工的生活补助。六是对城镇大集体困难企业中未参加养老保险、且基本生活保障水平低的退休职工，按每人每月200元的标准给予生活补助。七是进一步改善农村敬老院设施。八是不断完善慈善机构，发展社会慈善事业，提高救助水平。

4、努力解决群众看病就医问题。着力办好9件实事：一是实现城镇居民基本医疗保险全覆盖。其中低保人员和重度残疾人100%参保，所需资金由财政全额承担。二是扩大城镇职工基本医疗保险覆盖面，资助8万名国有及国有控股困难企业职工参保。三是新型农村合作医疗制度覆盖到全市12个县（市、区），参合人数达到454万人，人均筹资标准由50元增加到100元，参合农民住院一次报账补偿率达到90%。四是照顾农村计划生育户，独生子女和二女结扎户家庭成员参加新型农村合作医疗的个人承担费用由财政负担。五是推进城乡医疗救助，救助资金使用率力争提高到80%，新增的50万农村低保对象全部纳入救助范围。六是进一步做好儿童疫苗免费接种工作。七是进一步加强社区卫生服务机构达标建设，推进城市居民公共卫生服务券发放工作，让更多的城镇居民享受到实惠。八是提高1－6级伤残军人和在乡红军老战士医疗保险资金统筹标准，每人每年不低于8000元。九是推进残疾人康复工程，为2000名贫困白内障患者实施复明手术,为800名贫困精神病患者提供药物医疗救助,为30名贫困肢残人装配假肢,为80名贫困聋儿提供言语康复训练,为1300名残疾人提供辅助器具，为30名智力和精神残疾人提供特殊康复和托养服务。

5、扎实推进济困助学。着力办好6件实事：一是为全市城乡97.5万名义务教育阶段学生免除学杂费，为90万名公办学校义务教育阶段学生免费提供教科书。二是对全市6万名义务教育阶段经济困难家庭寄宿学生补助生活费。三是为普通高中学校经济困难家庭学生提供政府助学金，资助范围从公办学校延伸到民办学校。四是为中等职业学校一、二年级学生提供国家助学金。五是为普通高等院校全日制本专科在校经济困难家庭学生提供国家助学金。六是扩大对考入大学的本市经济困难家庭学生的资助范围，将考入民办高校和独立学院的经济困难家庭学生纳入资助范围。

6、加强廉租房和经济适用房建设。着力办好3件实事：一是扩大廉租房供应。全市新建廉租房不低于800套，其中中心城区不低于300套，全市所有县城廉租房补贴保障范围由人均 6平方米 提高到 8平方米 、由低保家庭扩大到城镇低收入家庭，做到应保尽保。二是继续推进经济适用房建设。经济适用房建设规模原则上按当地商品房开发总量的20%核定，力争新建经济适用房30万平方米，受益户数4400多户。三是积极探索微利商品房的建设、供应办法，逐步解决部分工薪阶层家庭购房困难。

7、加大扶贫开发力度。着力办好5件实事：一是对329个扶贫开发工作重点村实施"整村推进"扶贫，覆盖贫困人口7万人。二是做好7个比照实施西部大开发政策县的移民搬迁扶贫工作。三是安排200万元贴息资金，支持产业化扶贫龙头企业。四是安排专项资金，完成1.2万名贫困地区劳动力转移培训。五是继续推进市直单位定点扶贫和党员干部与贫困户结对帮扶工作。

8、切实改善群众的生产生活条件。着力办好6件实事：一是继续做好农村离任老村支书、老主任生活补助。二是安排3700多万元解决20多万农村人口安全饮水问题。三是新增农村沼气2.5万户。四是为20户以上、已通电自然村开通广播电视。五是为180个行政村接通数字网络宽带。六是安排专项资金为农民和农村学生免费放映电影。

（三）稳步推进基础设施建设

基础设施建设作为推进经济增长和社会发展的重要物质条件，事关上饶经济社会可持续发展的战略大局，必须坚持不懈地抓好。

加强交通基础设施建设。加快推进上饶三清山（赣东）机场前期工作。开工建设上武高速公路，积极配合昌德高速公路建设；加快国、省道公路和市重点道路工程建设，积极筹备320国道上饶段重建工作，力争年内开工，两年完成；完成农村公路硬化 1580公里 ，开工建设渡改桥项目15座。力争开工建设万年综合性码头和余干黄金埠电煤专用码头。积极做好九景（婺）衢铁路工程项目前期准备工作。抓紧制定规划，加快公交基础设施建设，优化运营结构，优先发展城市公共交通。市、县（区）联动，财政投入700万元，用于中心城区公交设施的改善。

加强农田水利基础设施建设。继续抓好第二期病险水库除险加固，积极做好第三期病险水库除险加固项目的申报工作；开工建设铅山伦潭水利枢纽工程；抓紧启动信州区沙溪堤、上饶县皂头堤等1-5万亩圩堤除险加固工程。继续实施好造地增粮富民工程，农业综合开发完成中低产田改造13万亩。继续抓好灌溉排涝工程建设，确保锣鼓山电排站、姚坊泵站更新改造在汛期前完工。抓好农业节水设施和技术推广。

加强能源基础设施建设。全面完成广丰、万年、德兴三县（市）220千伏输变电工程建设，确保上饶500千伏输变电工程在6月份建成投运，开工建设铅山220千伏输变电工程和鄱阳凯迪生物质发电项目，争取开工建设余干黄金埠电厂二期工程，争取立项建设鄱阳莲湖风电厂。

加强信息化基础设施建设。继续推进上饶市网上办公系统建设，努力实现无纸化办公，运行维护好"中国上饶"政府门户网站，扎实做好"全国农村信息化综合信息服务试点"工作，着力抓好上饶移动G网、上饶联通C网等工程建设。力争年末固定电话装机数达到112万部，移动电话装机数达到252万部，互联网用户达到31万户。

（四）加快城镇化和区域经济发展

坚持把实施城镇化战略作为促进区域经济发展、全面建设小康社会的一项重点工作来抓。

着力提升中心城区承载力。按照重大基础设施优先、产业引导与空间集聚原则，加快中心城区市政基础设施、公共配套设施建设。在城市道路交通方面：开工建设庆丰路、明淑路改造工程，实施信江大桥加固改造工程，开工建设丰溪大桥；加快建设广信大道、志敏大道、滨江西路；完成五三大道东延伸段、凤凰大道东段、三清山大道东延伸段工程；开工建设上饶汽运东、西客站，实施三江汽运客站改造工程。在公园绿化方面：启动云碧峰森林公园二期、龙潭公园、槠溪湿地公园、槠溪河东岸景观和上饶集中营景区绿化工程建设；完成中央公园、紫阳公园、旭日公园建设。在功能设施配套方面：开工建设奥林匹克中心、广电中U；启动建设上饶经济开发区工业污水处理厂；开工建设城东20万吨自来水厂，加快中心城区引水工程建设前期工作；完成中心城区8个公厕、7个垃圾中转站建设；推进龙潭区块整治改造工程；加快中心城区救助站建设，完成中心城区福利院工程，继续抓好信江书院修缮工作。在城市管理方面：建立健全城市管理长效机制，继续推进五城同创，积极做好市中心城区创建国家园林城市的各项基础工作，加大各县（市）创建国家、省级园林城市力度。按计划、分步骤地推进城市管理"数字化"建设，把城市管理纳入科学规范、运转有序的轨道。

继续做大城市产业。以上饶经济开发区为平台，重点打造机械加工、有色金属和光伏产业基地，力争年内实现主营业务收入200亿元、利税15亿元。以江南商贸城、渡口批发市场、赣东北农产品批发市场、万福广场等大商场（市场）为支撑，进一步形成布局合理的现代商贸流通体系。

不断壮大县域经济。抓紧编制主体功能区规划，优化经济布局，以产业为基础，以经济联系为纽带，以资源要素优化配置为依据，有序推进小城市群和产业带建设。继续发挥招商引资的助推作用，依托工业园区发展工业，依托骨干企业发展配套产业，依托优势资源发展特色产业，全面提升县域经济规模和水平。力争财政总收入县县超3亿元。

加快发展乡镇经济。科学编制中心城镇建设规划，切实加强乡镇的社会管理和公共服务职能。以工业园区吸纳就业为主，加快推进农村人口向城镇转移，培育小城镇的产业基础和经济基础，把一批带园区、景区、街区、矿区的乡镇建设成为具有较强辐射带动能力的中心镇。

（五）深入推进改革开放

做到好字优先，最根本的还是要深化改革，扩大开放，在完善体制机制上取得新突破，进一步增强经济社会发展的源动力。

加大改革攻坚力度。继续推进国企改革。抓好朝阳磷矿政策性破产工作，抓好企业依法改制工作。扎实推进国有资产管理体制改革。组建上饶市国有资产经营公司，稳步推进市直行政事业单位经营性资产统一经营管理工作。大力发展资本市场，积极做好企业股改上市。推进上饶汽运集团、远泉集团、三清山旅游集团和富达铜材上市进程；加快市本级中小企业信用担保公司增资扩股步伐。全面深化农村改革。实施好国有农场税费改革，积极稳妥地推进乡镇机构改革、县乡财政管理体制改革，深化林业产权制度配套改革。实施好公务员法的各项配套法规，平稳推进事业单位人事制度改革。

全面提高开放水平。坚持内外并举、"三外"并重的方针，抓住国家加工贸易政策调整的有利时机，以上饶经济开发区为龙头，以各县（市）工业园区为支撑，加大招商引资和区域合作力度，进一步扩大开放规模，拓宽开放领域，提升开放层次。一是创新招商方式。变招商引资为择商选资，变被动承接为主动对接，变单个项目对接为产业、区域对接，形成要素集聚和产业集聚。围绕大企业、大集团、大财团、大项目，全力攻坚，引进一批创新能力强、拥有自主品牌的高质量项目。着力推进与香港运科集团项目、台湾自行车产业园、人民电器产业园、鞋业袜业和电子电器基地等重大项目的洽谈和签约。二是强化平台建设。加强"海峡西岸网洽会·上饶馆"的宣传、维护和管理工作，加快建设南昌海关上饶办事处，确保年底封关运行。开工建设上饶"无水港"，确保年底投入使用。加快口岸"大通关"建设，完善"属地申报、口岸验放"等通关便利措施，使上饶成为江西距离出海口最近的城市。积极做好申报"中西部加工贸易重点承接地"工作，启动上饶出口加工区前期工作。三是积极稳妥地实施"走出去"战略。充分利用"两个市场、两种资源"，支持有条件的企业赴境外地区投资办厂和从事境外加工贸易，积极鼓励企业到境外开发利用战略性资源，扩大对外承包工程规模，提高对外劳务合作层次。确保年内实际利用外资达到2.8亿美元，增长12％；引进5000万元以上的省外内资工业项目实际进资达到104亿元，增长30％；完成外贸进出口3.3亿美元，增长17.9％；力争对外投资企业达到16家，完成对外承包工程业务营业额360万美元。

大力发展非公有制经济。毫不动摇地鼓励、支持和引导非公有制经济发展，让创业者在政策上有依据、法律上有保障、舆论上有支持、社会上有声誉，着力形成政府鼓励创业、百姓自主创业的浓厚氛围。鼓励民营企业以独资、合作、合资、联营、参股、特许经营等方式，参与国有企业改革、改组和改造，实现二次创业；进一步增强民营企业社会责任感，鼓励民营企业采取直接帮扶、项目外包、供应原料等方式带动百姓创业；鼓励下岗职工、退伍转业军人和高校毕业生自主创业；鼓励外出务工人员返乡创业。继续发挥工商联、行业协会、商会等组织的桥梁纽带作用，切实保护好民营企业的合法权益，促进民营企业健康发展。

（六）大力建设生态文明

优良的生态是上饶人民引以自豪的品牌。建设生态文明不仅关系到上饶的今天，更关系到上饶的未来。各级政府和部门一定要树立前瞻思维和长远眼光，坚持既要金山银山，更要绿水青山；既要经济效益，更要社会效益和生态效益，依靠全市人民的共同努力，建设和保护我们美丽的家园。

抓好资源节约和综合利用。以促进高耗能企业节能，推进房屋建筑土地(小区)节能试点和政府建筑物、办公系统节能改造为重点，着力抓好13家资源综合利用企业、12家清洁生产企业、9家再生资源回收和利用企业，大力发展循环经济，推进节能节材和资源再利用。强化各级政府土地管理和耕地保护责任制，加强对基本农田保护区的保护。认真实施土地开发整理项目，确保全市耕地总量稳定。积极抓好矿产资源合理开发和综合利用。

抓好节能减排。进一步强化目标责任制。把节能减排任务量化细化，纳入经济社会发展统计监测评价体系层层加以落实，切实把节能减排作为调整经济结构、转变发展方式的突破口抓紧抓好。进一步强化源头控制。禁止引进不符合国家产业政策、生产工艺落后、污染严重的项目，所有新建项目必须严格执行环境影响评价和"三同时"制度。加快淘汰落后产能。坚决取缔高耗能、高耗水、高耗材企业，对污染治理不能稳定达标或超出排放总量的企业，严令整改，直至关闭。进一步强化环境监测手段，不断健全在线监测网络，着力提高监测水平。进一步提高污水处理能力，加大对已投入使用的城市污水处理厂和污染治理设施的监管力度，完成婺源、德兴、玉山、广丰、万年、鄱阳六个县（市）污水处理厂建设，切实提高污染物的处理率和削减率。充分发挥排污收费的经济杠杆作用，用经济手段促进企业节能减排。继续加大信江、饶河流域和鄱阳湖区环境保护，突出抓好饮用水源保护区监管，确保饮用水源水质安全。

推进绿色创建工作。组织实施好长防林、退耕还林、血防林等林业重点工程，确保完成50万亩造林计划。加大水土流失整治力度，确保完成水土流失综合治理240平方公里。加强森林公园建设，力争新建1个国家级森林公园、2个省级森林公园。支持鄱阳县申报鄱阳湖国家湿地公园。

（七）全面推进社会各项事业

促进社会各项事业协调发展，是构建和谐社会的必然要求，是提高人民生活水平、文化素质、健康素质的根本需要，是发展成果由人民共享的具体体现。

大力推进科技进步。推进农业科技示范园区建设，组织实施好粮食丰产工程、绿色农业关键技术研发、制造业信息化和山江湖可持续发展试验区建设四个专项，争取申报1－3个国家级示范基地。扶持10家技术含量高、市场前景好的高新技术企业发展。推进企业与高等院校和科研院所联合。加强技术引进和推广应用。加强专业技术、经营管理、农村实用等各类人才队伍建设，继续在全市范围内深入推进农村小康科技特派员制度。

加快发展教育事业。统筹发展义务教育、高中阶段教育、高等教育，大力发展职业教育，注重发展幼儿教育和特殊教育，发展和规范民办教育。继续深化义务教育经费保障机制改革，切实加强农村薄弱学校建设，提高农村教育水平。整合中心城区高中教育资源，组建上饶中学。积极筹建上饶市特殊教育学校，开工建设上饶卫校新校区。

繁荣文化体育事业。不断夯实文化基础设施，完成市图书馆建设和821台搬迁。加快构建公共文化服务体系，重视城乡文化协调发展，精心组织上饶改革开放30周年摄影图片展等特殊文化活动，着力丰富农村和偏远地区群众的精神文化生活，建成一批农村乡镇综合文化站。大力发展文化产业，加快建设赣东北图书城等重点产业项目。加E网络文化建设，营造良好的网络环境。重视文物和非物质文化遗产保护。积极开展"全民健身与奥运同行"活动，举办市第三届老年运动会，推动体育事业全面发展。

提高人民健康水平。继续推进村卫生所、中心卫生院、县级综合医院、中医院、疾控中心实验室服务能力达标建设，实施"名院、名科、名医"工程，加快医疗卫生机构现代化建设。不断完善重大疾病防控体系，提高突发公共卫生事件应急处置能力。加快建设市麻风病康复中心，积极实施"血防百村攻坚行动"，扎实抓好血防综合防治工作。鄱阳、余干两个县达到疫情控制标准，玉山、上饶两个县达到传播阻断标准。

切实加强人口计生工作。坚决稳定低生育水平，着力提高出生人口素质，统筹解决人口问题。重点抓好农村的计划生育工作和流动人口的计划生育管理，以及党员干部、职工违法生育的清查处理，着力整治出生人口性别比偏高问题，力争用2-3年时间，把出生人口性别比降到全国平均水平。

高度重视安全生产和社会稳定。严格落实社会治安综合治理领导责任制，继续推进平安创建活动及和谐社区建设，切实加强流动人口管理，不断加快城乡社区警务改革，持续加大公共安全投入，着力构筑社会治安综合防控体系。深入开展专项整治斗争，突出打黑除恶、反盗防窃、扫黄打非三大重点，切实维护社会公共安全。进一步加强信访工作，加大矛盾纠纷排查调处力度，将社会不稳定因素化解在基层和萌芽状态。切实加强安全生产工作，进一步强化安全生产政府监管责任和企业主体责任，推进企业安全生产标准化建设，突出抓好重点行业、重点领域的专项治理，及时排查和消除重大安全隐患。加强防汛抗旱、畜禽疫病防控和森林防火工作，切实抓好3个山洪地质灾害监测预警系统项目建设，有效遏制重特大安全事故的发生。进一步健全应急管理机制，完善应急预案，提高应急处置能力。

切实加强国防和民兵预备役工作，重视和支持工会、共青团、妇联等群众性团体工作，关心妇女、儿童、老龄事业，积极发展残疾人事业和慈善事业，认真做好民族、宗教、对台、外事、侨务、防震减灾、人防、气象、档案、新闻出版、保密和地方志等工作。

三、切实加强政府自身建设

我们将认真学习贯彻党的十七大精神，进一步加强政府自身建设，不断提高行政能力，努力打造公信力高、执行力强、工作作风硬、群众信得过的政府。

推进依法行政，维护社会公平正义。进一步加强与人大和政协的联系，自觉接受人大及其常委会的法律监督和政协的民主监督。不断加强政府法制工作，以完善政府规范性文件备案审查和定期清理制度为重点，切实搞好行政机关内部监督。严格落实行政执法责任制，加大评议考核和责任追究力度，明确行政执法权限，完善行政执法程序，坚决防止多头执法、执法不公、执法违法等现象，确保各项行政权力在法治的轨道上公开、透明、规范运行。

切实转变职能，强化社会管理和公共服务。积极培育并强化公共财政理念，不断增强社会管理和公共服务意识，进一步创新政府管理经济的方式，真正把企业生产经营的投资决策权交给企业，把社会自我调节的职能交给中介机构，把群众自治范围内的事情交给群众自己依法办理。集中精力为市场主体创造良好的发展环境，为社会和市民提供优质的公共产品。不断提高政府管理社会和公共服务的能力。

规范行政行为，提高行政效能。进一步加强行政执法部门建设，不断健全部门间协调配合机制，减少和规范行政审批。不断创新重点项目服务制度，切实提高审批效能。着力解决职责交叉、政出多门问题，减少行政层次，降低行政成本。不断完善各类公开办事制度，建立健全质询、问责、经济责任审计制度。加强政务督查和效能监察，严格政府工作目标管理，强化工作落实机制，提高落实能力，确保政令畅通。

改进工作作风，勤政廉政为民。坚持以求真务实的作风推进各项工作，多干打基础、利长远的事。加强调查研究，倾听群众呼声，多为群众办好事、办实事。改进政府工作流程，简化办事程序，精简会议、改进文风，提高实效。加强公务员队伍建设，不断提高各级政府的理解力、执行力和操作力。严格执行党风廉政建设责任制，切实加强公务员队伍的廉洁自律工作，坚决纠正损害群众利益的不正之风，倡导勤俭节约，反对奢侈浪费，不断提高群众对政府的信任度和满意度。

各位代表，党的十七大为我们绘制了宏伟的发展蓝图。形势催人奋进，事业任重道远。让我们在中共上饶市委的正确领导下，紧紧依靠全市人民，万众一心，开拓奋进，共同创造上饶更加美好的未来。
